# Wet synthesis endows scalable epitaxy of complex oxide multiferroics

**DOI:** 10.1093/nsr/nwaa060

**Published:** 2020-04-08

**Authors:** Hua Zhou

**Affiliations:** X-ray Science Division, Advanced Photon Source, Argonne National Laboratory, USA

Synthesis is the bridge linking scientific fantasies to practical realizations, to push the boundaries of materials exploration with societally impactful applications. As one of the most promising classes of materials with direct technological implications, complex oxides exhibit an amazing collection of electronic, chemical, ionic and topological properties, making them strategic candidates for a variety of electronic, energy and environmental technologies [1]. Tremendous advances in thin-film growth technology over the past decades have led to effective control of structures and compositions during deposition so that even individual atomic layers can be robustly manipulated, for example by molecular beam epitaxy or pulsed laser deposition. Although the physical vapor deposition (PVD) techniques enable high-quality epitaxy, this has not been translated into large-scale synthesis with high efficiency for practical implementation of complex oxides in devices and system-level architectures. With respect to scalable synthesis, wet-chemistry-based growth methods such as sol-gel dip/spin/spray/blade coating have been demonstrated as technically viable strategies [2]. Nevertheless, most solution-based processing so far reported is inadequate to attain well-defined single-crystalline quality epitaxy with large area uniformity as most of the claimed ‘epitaxy growth’ was either weakly textured [3] or offered mundane discrete islands [4].

**Figure 1.**
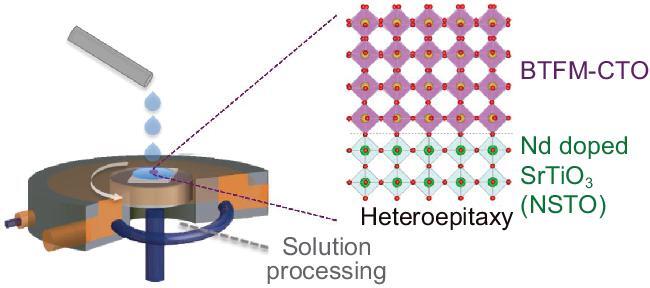
Schematic of multiferroic complex oxide epitaxy endowed by solution processing. Single-crystalline quality epitaxial BTFM–CTO films with thickness uniformity and surface atomic flatness up the centimeter scale can be synthesized using sol-gel solution processing (e.g. spin coater), effectively mitigating the evaporation rate during gelation. A large-scale epitaxial BTFM–CTO film exhibits room temperature ferroelectricity and bulk ferromagnetism, which enables large magnetically induced polarization switching. NSTO: Nd doped SrTiO_3_.

A recent work on wet synthesis of complex multiferroic oxides [5] exemplifies a compelling case of significant advances in synthesis of multifunctional materials with fascinating electronic and magnetic properties toward lab-to-manufacturing translation. The new experimental work, led by Prof. Jiangyu Li and collaborators, reports development of solution-based single-crystalline epitaxy in a large area, in particular tackling a complex oxide multiferroic system with its composition as sophisticated as (1-x)BiTi_(1-y)/2_Fe_y_Mg_(1-y)/2_O_3_–(x) CaTiO_3_ (BTFM–CTO), compared to simpler oxides in previous endeavors [4]. Using sol-gel solution processing and effectively mitigating the evaporation rate during gelation, as illustrated in Fig. 1, the authors achieved single-crystalline quality epitaxial BTFM–CTO films with thickness uniformity and surface atomic flatness up the centimeter scale. They employed a full suite of structural characterizations including X-ray thin film diffraction (e.g. for phase purity and epitaxial pseudomorphism) and cross-sectional scanning transmission electron microscopy (e.g. EDS and HAADF atomic profile for a well-defined abrupt interface and lattice registry), strictly verifying the impressive single crystalline quality of the epitaxial growth typically only accessible by PVD.

With the obtained BTFM–CTO epitaxial films, Li's team further confirmed the ferroelectricity resulting from strong spontaneous polarization and the bulk ferromagnetism at room temperature, compared to those of an archetypical BiFeO_3_ system, by quantifying the polarization vector, visualizing the domain switching and determining the magnetization moment. Moreover, non-volatile magnetically coupled polarization switching with a large coupling coefficient potential for magnetoelectric devices was illustrated in the phase-pure thin film by lateral piezoresponse force mapping of the domain pattern evolution under opposite in-plane magnetic fields.

The encouraging experimental work by Li's team substantiates that single crystalline quality heteroepitaxy with atomic precision of complex oxide materials bearing intricate compositions and subtly varied structures can be steadily and efficiently delivered in a large area via solution-based processing toward scalable manufacturing. Exciting exploration of new multiferroic epitaxial thin films through the synergy of wet synthesis and combinatory mining in a very wide composition- and strain-modulated phase space is envisaged.
